# Predictors and Correlates of Loneliness and Social Isolation in People With Dementia: Longitudinal Findings From the IDEAL Programme

**DOI:** 10.1002/gps.70191

**Published:** 2026-01-18

**Authors:** Isla Rippon, Christina R. Victor, Laura D. Gamble, Anthony Martyr, Catherine Quinn, Fiona E. Matthews, Linda Clare, the IDEAL programme team

**Affiliations:** ^1^ Department of Health Sciences College of Health Medicine and Life Sciences Brunel University of London Uxbridge UK; ^2^ Population Health Sciences Institute Newcastle University Newcastle UK; ^3^ NIHR Policy Research Unit in Dementia and Neurodegeneration University of Exeter (DeNPRU Exeter) Exeter UK; ^4^ National Drug and Alcohol Research Centre (NDARC) UNSW Medicine & Health Randwick Campus UNSW Sydney Australia; ^5^ REACH: The Centre for Research in Ageing and Cognitive Health University of Exeter Medical School University of Exeter Exeter UK; ^6^ Centre for Applied Dementia Studies University of Bradford Bradford UK; ^7^ Wolfson Centre for Applied Health Research Bradford UK; ^8^ Institute for Clinical and Applied Health Research Hull York Medical School University of Hull Hull UK; ^9^ NIHR Applied Research Collaboration South‐West Peninsula Exeter UK

**Keywords:** community, cultural activities, neighbourhood

## Abstract

**Objective:**

To identify predictors of loneliness and social isolation experienced by people with dementia at baseline and over time.

**Methods:**

Using data from the Improving the experience of Dementia and Enhancing Active Life (IDEAL) cohort study (2014–2018), we examined the prevalence and predictors of loneliness and social isolation in 1547 people with mild‐to‐moderate dementia over 24 months. Loneliness was measured using the six‐item De Jong Gierveld Scale at baseline and 24 months and social isolation by the six‐item Lubben Social Network Scale at baseline, 12 and 24 months. Generalised linear mixed effects models examined possible predictors of loneliness and social isolation including individual characteristics, depression, cognition, cultural participation, and neighbourhood characteristics.

**Results:**

At baseline 35.4% of people with dementia were categorised as being lonely and 28.8% as socially isolated, increasing to 39.3% and 32.0% 2 years later. Over the 24‐month follow‐up none of these predictors were associated with changes in social isolation scores. Only perceived neighbourhood trust was associated with change in loneliness longitudinally. At baseline, depressive symptoms, living alone, smaller social networks and lower neighbourhood trust were associated with greater loneliness. Cross‐sectionally, loneliness and lower cognitive ability were associated with greater social isolation, and greater cultural participation, more green and blue spaces nearby and higher neighbourhood trust were associated with lower social isolation scores.

**Conclusions:**

The findings highlight the importance of the local environment and cultural participation for people with dementia. Enhancing interactions with the local neighbourhood through initiatives such as dementia friendly communities may help to reduce loneliness and social isolation.

## Key Points

- We investigated the prevalence and predictors of loneliness and social isolation in a large cohort of people with dementia over a 24‐month period.
- The proportion of people who could be categorised as lonely or social isolated increased over the 24‐month period.
- People with dementia who lived alone, had higher depression scores and were more socially isolated were at a greater risk of loneliness at baseline.
- Increased participation in cultural activities, higher perceived availability of green or blue spaces and higher neighbourhood trust were associated with less social isolation at baseline.
- Only perceived neighbourhood trust was associated with change in loneliness longitudinally.

## 1 Introduction

The recent flagship report from the World Health Organization's Commission on Social Connection highlighted the risks to health and well‐being posed by loneliness and social isolation and identified the need to find ways to enhance social connections and reduce loneliness and social isolation. [1] Loneliness is defined as the discrepancy between expectations of quantity and quality of relationships and actuality. [2] Loneliness is an evaluative concept which can only be assessed subjectively by individuals while social isolation is characterised as a more objective measure of social connectedness with family, friends and the wider community. The prevalence of loneliness in older people in the general population in Western countries ranges from 19% to 34%. [3] Similarly, the prevalence of social isolation reported in the general population of older adults varies across studies, reflecting differences in measures and populations studied. A recent meta‐analysis of social isolation amongst community‐dwelling older adults estimated a prevalence of 22% (95% CI 14.0–40.0) in the European region. [4].

Both loneliness and social isolation have been identified as potential risk factors for dementia or cognitive impairment and other negative physical and mental health outcomes. [5, 6, 7, 8, 9] Our work complements this literature by focussing upon the experience of loneliness and social isolation amongst people with dementia and how this compares with their peers without dementia. Loneliness and social isolation have a negative impact on the life satisfaction and well‐being of people with dementia. [10, 11] Prior research on the experience of loneliness or social isolation from the perspective of people with dementia is limited, predominantly cross‐sectional [10, 11, 12, 13, 14, 15, 16, 17] and is largely focussed upon on loneliness and social isolation as risk factors for cognitive decline. [1, 18] Existing evidence addressing this question suggests that people with dementia experience comparable levels of loneliness to the general population of older people [14, 17] but greater social isolation. [11] Longitudinal research with people with dementia suggest that levels of social isolation may increase over time. [13] For example, an 18 month longitudinal study of 464 people with mild‐to‐moderate Alzheimer's disease reported that mean social network scores decreased by one point. [13] Longitudinally greater dementia severity was associated with social network deterioration (rather than vice versa). We found no studies reporting changes in loneliness longitudinally.

Studies of older people in the general population have identified female sex, living alone, lower levels of education and not being married as risk factors of loneliness. [19, 20] Characteristics of the local environment, such as low sense of belonging and trust in the neighbourhood are also predictive of loneliness. [21] Reviews of social isolation in older people have identified several factors as associated with social isolation: increased age; male sex; socio‐economic factors; poor mental health; declining cognitive function; life events or changes in work and family roles (including retirement); and environmental factors (including living arrangements, housing, geographic location or accessibility of the local neighbourhood or community). [22, 23, 24] Perceived neighbourhood cohesion or quality and social trust may influence social isolation, [25] as well as participation in social and cultural activities. [22] Reviews of correlates of and risk factors for loneliness and social isolation in older people highlight the need for the use of consistent and validated measures, in particular, for social isolation. [26, 27] We included the De Jong Gierveld Loneliness Scale [28] and the six‐item Lubben Social Network Scale, [29] two well established measures of loneliness and social isolation.

Given the very limited longitudinal evidence focussed on how loneliness and social isolation in people with dementia changes over time the aims of our study were to build upon our previous cross‐sectional analysis of loneliness [14] to: (a) examine the prevalence of loneliness and social isolation in people with dementia longitudinally; (b) identify risk factors for social isolation in people with dementia; and (c) identify risk factors that predict change in loneliness and social isolation over 2 years.

## 2 Materials and Methods

### 2.1 Design and Sample

We used data from the Improving the experience of Dementia and Enhancing Active Life (IDEAL) programme, [30] a longitudinal cohort study involving community‐dwelling people with dementia and carers in Great Britain. Participants with dementia and their respective carers were recruited through 29 National Health Service (NHS) Clinical Research Network sites throughout England, Scotland and Wales between July 2014 and August 2016. The inclusion criteria required that people with dementia have a clinical diagnosis of dementia (any type), which was in the mild‐to‐moderate stage as indicated by a Mini‐Mental State Examination (MMSE) [31] score of 15 or over, and to be living in the community at the time of enrolment. Exclusion criteria were a comorbid terminal illness, inability to provide informed consent, and any known potential for home visits to pose a significant risk to researchers (see study protocol for full details). [30].

We used baseline IDEAL data (T1: 2014–16) and follow up data at 12 months (T2: 2015–17) and 24 months (T3: 2016–18). The total number of participants at baseline (T1) was 1,537, and it decreased to 1183 at T2 and to 851 at T3.

Information was collected by direct interview from people with dementia who were visited at home by a researcher on three separate occasions. During the initial visit eligibility was checked and consent was obtained. Visits were up to 2 hours in length and interview time was shorter at T2 and T3 in comparison to T1. Participants received a modest payment in the form of a shopping voucher upon completion as a thank you for their involvement. For the current study we used a sub‐sample of measures included in the IDEAL study. The IDEAL sample is comparable with general population estimates for the proportion of people in each dementia sub‐type, with Alzheimer's disease being the most common diagnosis and more so amongst women.

The IDEAL study was approved by the Wales Research Ethics Committee 5 (reference 13/WA/0405) and the Ethics Committee of the School of Psychology, Bangor University (reference 2014–11684). The IDEAL study is registered with UKCRN, registration number 16593.

### 2.2 Measures

*Loneliness* was measured using the revised six‐item version of the De Jong Gierveld Loneliness Scale. [28] Total scores range from 0 to 6 with higher scores indicating more severe loneliness. The threshold for moderate loneliness is a score of 2–4 with severe loneliness defined as score of 5 or 6. [32] Data on loneliness was not collected at T2.

*Social isolation*: the six‐item Lubben Social Network Scale was used to measure gauge social isolation defined as perceived social support received from family and friends.^29^ Total scores ranged from 0 to 30 where a lower score is seen to indicate a higher risk of social isolation. Respondents with a score below 12 are, by convention, categorised as socially isolated.

*Depressive symptoms* were assessed using the 10‐item Geriatric Depression Scale (GDS‐10), [33] with higher total scores signifying greater depressive symptoms.

*Cultural participation* was assessed by ratings of frequency of engagement in 13 cultural activities such as going to the theatre, visiting museums, playing bingo, visiting stately homes or historic sites and going out to eat, [34] with higher scores indicating greater cultural engagement. Possible scores ranged from 13 (never took part in any of the activities) to 65 (took part in each activity more than once a week).

*Green and blue spaces:* this item asked participants about the availability of 12 types of green (e.g., parks, woodlands) and blue (e.g., lakes, rivers) spaces within a ten‐minute walk of where they live and was adapted from a ‘yes’ or ‘no’ question that was included in the National Survey for Wales. [35] The total number of local green and blue spaces was summed.

*Neighbourhood reciprocity and trust* was assessed by asking the participants to estimate the likelihood of a lost purse or wallet being returned with nothing missing. [36] Responses were grouped into ‘likely’ and ‘not likely/don't know’.

*Deprivation in the local area* was assessed using the Index of Multiple Deprivation (IMD). IMD is an official measure of relative deprivation for small areas or neighbourhoods in Great Britain. [37] It combines information from seven domains (income; employment; education, skills and training; health and disability; crime; barriers to housing and services; and living environment) to produce an overall relative measure of deprivation. England, Scotland and Wales each have their own version of the IMD. The index was divided into quintiles to facilitate comparison of deprivation levels across the three countries. Scores are presented in quintiles ranging from most deprived to least deprived area.

Demographic information was collected covering age, sex, education (based on highest qualification achieved) and living situation (whether they lived alone or not). The specific dementia diagnosis was recorded from medical notes and cognitive function was assessed using the MMSE. [31].

### 2.3 Statistical Analysis

Descriptive statistics are reported for the total sample at T1‐T3 alongside those who completed the loneliness (T1 and T3) and social isolation measures (T1‐T3). Mixed effects regression analyses were used to investigate change in loneliness and social isolation measured over the three timepoints of data collection (T1‐T3) and also the influence of other variables on this change. Models with a random intercept were used and all models had unstructured covariance allowing subject‐specific random slopes to vary freely over time. For loneliness mixed effects models with a gamma distribution were used and estimate the rate ratios (RR) for the intercept and slope. An unadjusted model was conducted followed by a series of models adjusting for age, sex, education and living situation (model 1); cognition and dementia diagnosis (model 2), cultural participation, perceived neighbourhood trust, perceived availability of green and blue spaces in the local area and level of deprivation in the local area (IMD) (model 3) and depressive symptoms (model 4). Model 4 for loneliness also included social isolation and model 4 for social isolation included loneliness. In both sets of models the outcome measures were continuous. All data were analysed using Stata 17 (StataCorp LP, College Station, TX). The analyses are based on version 7.0 of the IDEAL datasets.

## 3 Results

Of the 1537 people with dementia participating at baseline, 1452 completed the social isolation measure (T1) and 1435 the loneliness measure. At T2 1087 respondents had social isolation data and at T3 736 had isolation data and 758 completed the loneliness measure (Table 1). Missing data on predictors ranged from 0.1% (living situation and MMSE test scores) to 10.9% for depressive symptoms. At baseline the majority of participants were aged 65 or over (91%), just under half (43%) were female and approximately 17% lived alone (Table 1). People with dementia perceived there to be an average of 6 types of green and blue space within a ten‐minute walk and three‐quarters thought that people in their local community would be likely to return a missing wallet or purse. Around 30% of participants lived in the least deprived areas, with under 10% living in the most deprived areas. At T1 35.4% of participants were classed as lonely and 28.8% as isolated, increasing to 39.3% and 32% respectively at T3 (Table 2). Over the 2‐year follow‐up mean loneliness scores remained stable while mean social isolation scores decreased indicating increasing social isolation.

**TABLE 1:** Sample characteristics of people with dementia at baseline, 12 and 24 months.

	T1			T2		T3		
	Total	Loneliness^a^	Social isolation^b^	Total	Social isolation	Total	Loneliness	Social isolation
	*N* = 1537	*N* = 1435	*N* = 1452	*N* = 1183	*N* = 1095	*N* = 851	*N* = 763	*N* = 741
	*N* (%)/Mean (SD)	*N* (%)/Mean (SD)	*N* (%)/Mean (SD)	*N* (%)/Mean (SD)	*N* (%)/Mean (SD)	*N* (%)/Mean (SD)	*N* (%)/Mean (SD)	*N* (%)/Mean (SD)
Sex								
Male	865 (56.3%)	810 (56.5%)	818 (56.3%)	669 (56.6%)	623 (56.9%)	476 (55.9)	434 (56.9%)	418 (56.4%)
Female	672 (43.7%)	625 (43.6%)	634 (43.7%)	514 (43.5%)	472 (43.1%)	375 (44.1%)	329 (43.1%)	323 (43.6%)
Age								
Under 65	134 (8.7%)	130 (9.1%)	131 (9.0%)	89 (7.5%)	87 (8.0%)	67 (7.9%)	63 (8.3%)	62 (8.4%)
65–69	177 (11.5%)	168 (11.7%)	170 (11.7%)	129 (10.9%)	123 (11.2%)	71 (8.3%)	66 (8.7%)	64 (8.6%)
70–74	258 (16.8%)	244 (17.0%)	239 (16.5%)	193 (16.3%)	176 (16.1%)	160 (18.8%)	139 (18.2%)	138 (18.6%)
75–79	366 (23.8%)	338 (23.6%)	348 (24.0%)	268 (22.7%	252 (23.0%)	171 (20.1%)	153 (20.1%)	143 (19.3%)
80+	602 (39.2)	555 (38.7%)	564 (38.8%)	504 (42.6%)	457 (41.7%)	382 (44.9%)	342 (44.8%)	334 (45.1%)
Mean age	76.4 (8.5)	76.2 (8.5)	76.2 (8.5)	77.2 (8.4)	77.0 (8.4)	77.5 (8.4)	77.4 (8.5)	77.5 (8.6)
Education								
No qualifications	429 (28.0%)	392 (27.4%)	415 (28.7%)	318 (27.1%)	301 (27.7%)	232 (27.3%)	206 (27.2%)	202 (27.5%)
School leaving certificate at age 16	272 (17.8%)	258 (18.0%)	258 (17.8%)	197 (16.8%)	183 (16.8%)	136 (16.0%)	123 (16.2%)	119 (16.2%
School leaving certificate at age 18	519 (33.9%)	492 (34.4%)	484 (33.5%)	410 (35.0%)	378 (34.8%)	295 (34.7%)	265 (35.0%)	265 (36.0%)
University	311 (20.3%)	288 (20.1%)	290 (20.4%)	248 (21.1%)	225 (20.7%)	182 (21.4%)	164 (21.6)	150 (20.4%)
Missing	6	5	5	10	8	6	5	5
Living situation								
Live with someone	1267 (81.3%)	1167 (81.6%)	1185 (81.6%)	958 (81.1%)	891 (81.3%)	687 (80.7%)	624 (81.8%)	603 (81.4%)
Live alone	288 (18.7%)	266 (18.5%)	265 (18.4%)	200 (16.9)	189 (17.4%)	134 (15.8%)	124 (16.3%)	123 (16.6%)
Living in care	0	0	0	24 (2.0%)	14 (1.3)	29 (3.4%)	14 (1.8%)	14 (1.9%)
Missing	2	2	2	1	1	1	1	1
Dementia diagnosis								
Alzheimer's disease	851 (55.4%)	424 (58.2%)	807 (55.6%)	664 (55.9%)	613 (56.0%)	488 (57.3%)	444 (58.2%)	426 (57.5%)
Vascular dementia	170 (11.1%)	73 (10.0%)	157 (10.8%)	116 (9.8%)	106 (9.7%)	82 (9.6%)	74 (9.7%)	72 (9.7%)
Mixed Alzheimer's disease & vascular dementia	324 (21.1%)	161 (22.1%)	305 (21.0%)	264 (22.3%)	243 (22.2%)	185 (21.7%)	165 (21.6%)	162 (21.9%)
Other diagnosis	192 (12.5%)	177 (12.3%)	183 (12.6%)	142 (12.0%)	133 (12.1%)	96 (11.2%)	80 (10.5%)	81 (10.9%)
Cognition (MMSE)								
Mean (SD)	23.2 (3.6)	23.2 (3.6)	23.2 (3.6)	21.6 (5.1)	22.0 (4.8)	20.5 (6.2)	21.2 (5.7)	21.5 (5.5)
Missing	1	1	1	12	8	12	7	6
Depression								
Mean (SD)	2.7 (2.3)	2.6 (2.3)	2.7 (2.3)	2.4 (2.3)	2.4 (2.2)	2.4 (2.1)	2.4 (2.1)	2.4 (2.1)
Missing	169	142	134	108	75	97	61	56
Cultural participation								
Mean (SD; range 13–44)	22.8 (5.6)	22.9 (5.6)	22.9 (5.5)	22.2 (5.5)	22.2 (5.4)	21.6 (5.4)	21.7 (5.4)	21.7 (5.4)
Missing	86	59	77	107	76	113	70	59
Neighbourhood trust								
Likely	1126 (75.7%)	1065 (75.9%)	1065 (75.7%)	881 (79.2%)	835 (79.3%)	589 (76.0%)	554 (77.1%)	538 (76.4%)
Not Likely/don't know	362 (24.3%)	339 (24.2%)	342 (24.3%)	232 (20.8%)	218 (20.7%)	186 (24.0%)	165 (23.0%)	166 (23.6%)
Missing	49	31	45	70	42	76	44	37
Green & blue spaces								
Mean (SD)	6.1 (2.7)	6.0 (2.7)	6.0 (2.7)	6.1 (2.7)	6.2 (2.7)	6.2 (2.7)	6.2 (2.7)	6.2 (2.7)
Missing	50	31	46	24	19	16	12	13
Index of multiple deprivation (IMD)								
Q1 (most deprived)	129 (8.4%)	120 (8.4%)	124 (8.5%)	91 (7.7%)	87 (8.0%)	68 (8.0%)	62 (8.2%)	62 (8.4%)
Q2	234 (15.2%)	226 (15.8%)	224 (15.4%)	181 (15.4%)	174 (16.0%)	133 (15.7%)	122 (16.1%)	121 (16.4%)
Q3	327 (21.3%)	298 (20.8%)	311 (21.4%)	238 (20.3%)	221 (20.3%)	171 (20.2%)	157 (20.7%)	153 (20.8%)
Q4	379 (24.7%)	355 (24.7%)	359 (24.7%)	305 (26.0%)	283 (26.0%)	219 (25.9%)	192 (25.3%)	194 (26.4%)
Q5 (least deprived)	468 (30.5%)	436 (30.4%)	434 (29.9)	360 (30.6%)	322 (29.6%)	255 (30.1%)	225 (29.7%)	206 (28.0%)
				8	8	5	5	5

^a^ Total sample for loneliness analysis.

^b^ Total sample for social isolation analysis.

**TABLE 2:** Prevalence of loneliness and social isolation.

	T1	T2	T3
Loneliness			
Not lonely (N,%)	936 (64.6%)		460 (60.7%)
Moderately lonely (N,%)	441 (30.4%)		260 (34.3%)
Severely lonely (N,%)	73 (5.0%)		38 (5.0%)
Mean (SD)	1.4 (1.5)		1.4 (1.5)
N	1435		758
Missing	102		93
Social isolation			
Isolated (N,%)	427 (28.8%)	328 (29.9%)	238 (32.0%)
Non‐isolated (N,%)	1058 (71.3%)	771 (70.2%)	506 (68.0%)
Mean (SD)	15.4 (6.2)	14.9 (6.2)	14.6 (6.2)
N	1452	1087	736
Missing	85	96	115

*Note:* Less missing data on categorical variables. Total participants at *T*1 = 1537, *T*2 = 1183, *T*3 = 851.

### 3.1 Correlates and Predictors of Loneliness at Baseline and Longitudinally

#### 3.1.1 Longitudinal

The unadjusted model showed a slight increase in loneliness scores between baseline and T3 (RR 1.02, 95% CI 1.00, 1.04; *p* = 0.029) but this was attenuated once socio‐demographic factors were considered (Table 3, model 1). In the final model (model 4), the only independent factor predictive of loneliness over time was perceived neighbourhood trust (RR 0.92; 95% 0.88–0.98; *p* = 0.005). Although the perceived lack of trust group had greater loneliness at baseline this group had a lower increase in loneliness score between T1 and T3 than the likely to trust group (0.5% vs. 7.0%).

**TABLE 3:** Models examining predictors of loneliness in people with dementia.

	Model 1		Model 2		Model 3		Model 4	
	Intercept	Slope	Intercept	Slope	Intercept	Slope	Intercept	Slope
	Rate ratio (95% CI)	Rate ratio (95% CI)	Rate ratio (95% CI)	Rate ratio (95% CI)	Rate ratio (95% CI)	Rate ratio (95% CI)	Rate ratio (95% CI)	Rate ratio (95% CI)
Loneliness	2.10 (1.94, 2.27)^***^	0.99 (0.94, 1.04)	2.21 (1.78, 2.74)^***^	1.03 (0.88, 1.20)	2.54 (1.96, 3.29)^***^	1.09 (0.91, 1.32)	1.99 (1.54, 2.57)^***^	1.11 (0.91, 1.36)
Sex								
Male	Ref.	Ref.	Ref.	Ref.	Ref.	Ref.	Ref.	Ref.
Female	0.96 (0.90, 1.03)	1.01 (0.97, 1.06)	0.97 (0.91, 1.04)	1.01 (0.97, 1.06)	0.97 (0.91, 1.03)	1.02 (0.97, 1.06)	0.96 (0.90, 1.02)	1.02 (0.97, 1.07)
Age								
80 and over	Ref	Ref.	Ref.	Ref.	Ref.	Ref.	Ref.	Ref.
Under 65	1.31 (1.17, 1.47)^***^	1.01 (0.94, 1.09)	1.31 (1.17, 1.47)^***^	1.00 (0.93, 1.08)	1.29 (1.14, 1.45)^***^	1.02 (0.94, 1.10)	1.18 (1.06, 1.33)^**^	1.02 (0.94, 1.11)
65–69	1.07 (0.97, 1.19)	1.03 (0.96, 1.10)	1.07 (0.96, 1.19)	1.02 (0.96, 1.09)	1.08 (0.97, 1.20)	1.03 (0.96, 1.10)	1.04 (0.94, 1.15)	1.03 (0.96, 1.11)
70–74	1.14 (1.04, 1.25)^**^	1.01 (0.95, 1.07)	1.14 (1.04, 1.25)^**^	1.01 (0.95, 1.07)	1.14 (1.03, 1.25)^**^	1.03 (0.97, 1.09)	1.09 (0.99, 1.19)	1.02 (0.95, 1.08)
75–79	1.02 (0.94, 1.10)	1.05 (0.99, 1.11)	1.02 (0.94, 1.10)	1.05 (0.99, 1.11)	1.02 (0.94, 1.11)	1.06 (1.00, 1.12)	0.99 (0.91, 1.07)	1.08 (1.02, 1.15)^**^
Education								
No qualifications	Ref.	Ref.	Ref.	Ref.	Ref.	Ref.	Ref.	Ref.
School leaving certificate at age 16	0.92 (0.84, 1.01)	1.01 (0.94, 1.08)	0.92 (0.84, 1.01)	1.01 (0.95, 1.08)	0.96 (0.87, 1.05)	1.02 (0.95, 1.09)	0.98 (0.89, 1.07)	1.02 (0.95, 1.09)
School leaving certificate at age 18	0.90 (0.83, 0.98)^*^	1.03 (0.97, 1.08)	0.90 (0.83, 0.98)^*^	1.03 (0.97, 1.09)	0.93 (0.86, 1.01)	1.03 (0.97, 1.09)	0.97 (0.89, 1.05)	1.04 (0.98, 1.11)
University	0.90 (0.82, 0.99)^*^	1.04 (0.98, 1.10)	0.91 (0.82, 1.00)^*^	1.04 (0.98, 1.11)	0.97 (0.88, 1.07)	1.05 (0.99, 1.13)	0.95 (0.87, 1.05)	1.05 (0.98, 1.12)
Living situation								
Live with someone	Ref.	Ref.	Ref.	Ref.	Ref.	Ref.	Ref.	Ref.
Live alone	1.30 (1.19, 1.41)^***^	0.96 (0.91, 1.02)	1.31 (1.20, 1.42)^***^	0.96 (0.91, 1.02)	1.31 (1.20, 1.42)^***^	0.95 (0.90, 1.00)	1.25 (1.15, 1.36)^***^	0.95 (0.90, 1.01)
Dementia diagnosis								
Alzheimer's disease			Ref.	Ref.	Ref.	Ref.	Ref.	Ref.
Vascular dementia			1.06 (0.96, 1.18)	1.04 (0.97, 1.11)	1.05 (0.95, 1.17)	1.02 (0.95, 1.10)	0.99 (0.90, 1.10)	1.04 (0.97, 1.12)
Mixed Alzheimer's disease & vascular dementia			1.06 (0.98, 1.15)	1.02 (0.97, 1.07)	1.04 (0.96, 1.13)	1.02 (0.97, 1.08)	0.99 (0.92, 1.07)	1.02 (0.97, 1.08)
Other diagnosis			1.16 (1.05, 1.28)^**^	1.07 (0.99, 1.15)	1.15 (1.04, 1.27)^**^	1.08 (1.00, 1.16)^*^	1.04 (0.94, 1.14)	1.10 (1.02, 1.18)^*^
MMSE			1.00 (0.99, 1.00)	1.00 (0.99, 1.00)	1.00 (0.99, 1.01)	1.00 (0.99, 1.00)	1.00 (0.99, 1.01)	1.00 (0.99, 1.00)
Cultural participation					0.99 (0.98, 0.99)^***^	1.00 (1.00, 1.00)	1.01 (1.00, 1.01)	1.00 (0.99, 1.00)
Neighbourhood trust								
Likely					Ref.	Ref.	Ref.	Ref.
Not Likely/don't know					1.16 (1.08, 1.25)^***^	0.94 (0.89, 0.99)^*^	1.08 (1.01, 1.17)^*^	0.92 (0.88, 0.98)^**^
Green & blue spaces					1.00 (0.99, 1.02)	1.00 (0.99, 1.01)	1.01 (1.00, 1.02)	1.00 (0.99, 1.01)
Index of multiple deprivation (IMD)								
Q1 (most deprived area)					Ref.	Ref.	Ref.	Ref.
Q2					0.96 (0.84, 1.10)	0.93 (0.85, 1.02)	0.95 (0.84, 1.08)	0.94 (0.86, 1.03)
Q3					0.94 (0.82, 1.06)	0.95 (0.87, 1.04)	0.95 (0.84, 1.08)	0.95 (0.87, 1.04)
Q4					0.95 (0.83, 1.07)	0.94 (0.86, 1.02)	1.00 (0.88, 1.13)	0.91 (0.84, 1.00)*
Q5 (least deprived area)					1.01 (0.89, 1.15)	0.96 (0.88, 1.05)	1.04 (0.92, 1.17)	0.97 (0.89, 1.06)
Depressive symptoms							1.09 (1.08, 1.11)^***^	1.00 (0.99, 1.01)
Social isolation							0.98 (0.97, 0.98)^***^	1.00 (1.00, 1.01)

Abbreviations: CI = Confidence Interval, Model 1: socio‐demographic factors (age, sex, education and living situation), Model 2: Model 1 + cognition and dementia diagnosis, Model 3: Model 2 + cultural participation, neighbourhood trust, green/blue spaces and deprivation in the local area (Index of Multiple Deprivation, IMD), Model 4: Model 3 + depressive symptoms and social isolation.

^*^ *p* < 0.05.

^**^ *p* < 0.01.

^***^ *p* < 0.001.

#### 3.1.2 Baseline

At T1, respondents with higher education were less lonely (Table 3, model 1) as were those with greater cultural participation and perceived access to blue and green spaces (model 3). There was no substantive evidence of an association between dementia diagnosis or cognition and loneliness at T1 or with level of deprivation in the local area (model 4). At baseline people with dementia who lived alone (RR 1.25; 95% CI 1.15, 1.36; *p* < 0.001) or were aged under 65 (RR 1.18; 95% CI 1.06, 1.33; *p* = 0.004) were more likely to be lonely (model 4) as were those who were more social isolated (RR 0.98; 95% CI 0.97, 0.98; *p* < 0.001) or had greater depressive symptoms (RR 1.09; 95% CI 1.08, 1.11; *p* < 0.001).

### 3.2 Correlates and Predictors of Social Isolation at Baseline and Longitudinally

#### 3.2.1 Longitudinal

In the unadjusted model there was a significant increase in social isolation across the 24‐month period −0.43 (95% CI ‐0.61, −0.25; *p* < 0.001), but this was attenuated once sociodemographic factors and living alone were considered (Table 4, model 1). No longitudinal risk factors were observed in the final model (model 4).

**TABLE 4:** Linear mixed models examining predictors of social isolation in people with dementia.

	Model 1		Model 2		Model 3		Model 4	
	Intercept	Slope	Intercept	Slope	Intercept	Slope	Intercept	Slope
	Estimate (95% CI)	Estimate (95% CI)	Estimate (95% CI)	Estimate (95% CI)	Estimate (95% CI)	Estimate (95% CI)	Estimate (95% CI)	Estimate (95% CI)
Social isolation	13.46 (12.67, 14.25)^***^	−0.21 (−0.68, 0.27)	9.65 (7.52, 11.78)^***^	−0.08 (−1.43, 1.27)	3.45 (0.95, 5.94)^***^	0.20 (−1.46, 1.86)	5.37 (2.74, 8.00)^***^	0.40 (−1.40, 2.20)
Sex								
Male	Ref.	Ref.	Ref.	Ref.	Ref.	Ref.	Ref.	Ref.
Female	−0.11 (−0.76, 0.54)	−0.09 (−0.47, 0.30)	0.00 (−0.65, 0.66)	−0.08 (−0.46, 0.31)	−0.02 (−0.66, 0.62)	−0.14 (−0.54, 0.25)	−0.06 (−0.69, 0.60)	−0.27 (−0.69, 0.16)
Age								
80 and over	Ref	Ref.	Ref.	Ref.	Ref.	Ref.	Ref.	Ref.
Under 65	−0.91 (−2.06, 0.23)	0.40 (−0.25, 1.06)	−1.09 (−2.25, 0.06)	0.41 (−0.26, 1.08)	−1.27 (−2.43, −0.12)^*^	0.52 (−0.18, 1.21)	−0.28 (−1.47, 0.90)	0.36 (−0.37, 1.10)
65–69	1.14 (0.10, 2.18)^*^	0.04 (−0.54, 0.62)	0.89 (−0.16, 1.94)	0.06 (−0.53, 0.54)	0.35 (−0.67, 1.36)	0.13 (−0.47, 0.72)	0.87 (−0.17, 1.90)	0.01 (−0.63, 0.64)
70–74	1.00 (0.08, 1.92)^*^	0.24 (−0.30, 0.77)	0.83 (−0.10, 1.75)	0.24 (−0.30, 0.78)	0.17 (−0.74, 1.08)	0.27 (−0.28, 0.82)	0.88 (−0.07, 1.83)	0.05 (−0.55, 0.65)
75–79	1.08 (0.27, 1.89)^**^	−0.12 (−0.60, 0.36)	1.00 (0.19, 1.81)^*^	−0.11 (−0.52, 0.37)	0.61 (−0.18, 1.40)	−0.08 (−0.57, 0.41)	0.74 (−0.09, 1.56)	−0.26 (−0.79, 0.26)
Education								
No qualifications	Ref.	Ref.	Ref.	Ref.	Ref.	Ref.	Ref.	Ref.
School leaving certificate at age 16	1.38 (0.45, 2.32)^**^	−0.34 (−0.90, 0.22)	1.18 (0.24, 2.12)^*^	−0.32 (−0.88, 0.24)	0.67 (−0.25, 1.60)	−0.40 (−0.98, 0.17)	0.56 (−0.39, 1.51)	−0.39 (−1.00, 0.23)
School leaving certificate at age 18	2.22 (1.41, 3.02)^***^	‐0.38 (‐0.85, 0.09)	2.00 (1.19, 2.80)^***^	‐0.36 (‐0.83, 0.12)	0.97 (0.17, 1.78)^*^	‐0.43 (‐0.93, 0.06)	0.62 (‐0.22, 1.46)	‐0.35 (‐0.88, 0.18)
University	1.83 (0.92, 2.75)^***^	‐0.30 (‐0.83, 0.23)	1.48 (0.56, 2.40)^**^	‐0.26 (‐0.80, 0.28)	‐0.32 (‐1.27, 0.64)	‐0.33 (‐0.91, 0.25)	‐0.55 (‐1.55, 0.45)	‐0.21 (‐0.84, 0.41)
Living situation								
Live with someone	Ref.	Ref.	Ref.	Ref.	Ref.	Ref.	Ref.	Ref.
Live alone	−0.79 (−1.63, 0.05)	−0.03 (−0.53, 0.46)	−0.91 (−1.75, −0.07)^*^	−0.03 (−0.53, 0.46)	−0.38 (−1.21, 0.45)	−0.01 (−0.51, 0.50)	0.40 (−0.46, 1.27)	−0.05 (−0.59, 0.50)
Dementia diagnosis								
Alzheimer's disease			Ref.	Ref.	Ref.	Ref.	Ref.	Ref.
Vascular dementia			−0.72 (−1.74, 0.31)	0.33 (−0.29, 0.99)	−0.38 (−1.39, 0.63)	0.48 (−0.15, 1.11)	−0.40 (−1.44, 0.65)	0.28 (−0.40, 0.96)
Mixed Alzheimer's disease & vascular dementia			−0.57 (−1.37, 0.22)	0.16 (−0.30, 0.62)	−0.26 (−1.04, 0.51)	0.14 (−0.33, 0.60)	0.04 (−0.77, 0.84)	−0.01 (−0.52, 0.49)
Other diagnosis			−0.14 (−1.11, 0.83)	0.12 (−0.48, 0.72)	0.09 (−0.85, 1.04)	0.04 (−0.56, 0.65)	0.36 (−0.63, 1.34)	−0.18 (−0.84, 0.47)
MMSE			0.18 (0.10, 0.27)^***^	−0.01 (−0.07, 0.04)	0.12 (0.03, 0.21)^**^	0.01 (−0.05, 0.06)	0.13 (0.04, 0.22)^**^	−0.01 (−0.07, 0.05)
Cultural participation					0.30 (0.24, 0.36)^***^	−0.00 (−0.04, 0.03)	0.27 (0.21, 0.33)^***^	−0.01 (−0.05, 0.03)
Neighbourhood trust								
Likely					Ref.	Ref.	Ref.	Ref.
Not Likely/don't know					−1.50 (−2.23, −0.78)^***^	0.21 (−0.24, 0.66)	−0.98 (−1.74, −0.22)^*^	0.20 (−0.30, 0.69)
Green & blue spaces					0.24 (0.13, 0.36)^***^	−0.08 (−0.15, −0.01)^*^	0.24 (0.12, 0.36)^***^	−0.05 (−0.13, 0.02)
Index of multiple deprivation (IMD)								
Q1 (most deprived area)					Ref.	Ref.	Ref.	Ref.
Q2					0.84 (−0.44, 2.11)	−0.48 (−1.26, 0.31)	0.47 (−0.82, 1.76)	−0.50 (−1.32, 0.32)
Q3					0.25 (−0.98, 1.48)	−0.13 (−0.90, 0.63)	−0.01 (−1.27, 1.25)	−0.25 (−1.05, 0.55)
Q4					0.84 (−0.38, 2.06)	−0.17 (−0.92, 0.59)	0.65 (−0.59, 1.90)	−0.22 (−1.01, 0.57)
Q5 (least deprived area)					0.70 (−0.51, 1.92)	−0.02 (−0.78, 0.74)	0.72 (−0.51, 1.95)	−0.10 (−0.90, 0.69)
Depressive symptoms							−0.04 (−0.20, 0.11)	−0.00 (−0.10, 0.10)
Loneliness							−1.14 (−1.37, −0.90)^***^	0.14 (−0.01, 0.30)

Abbreviations: CI = Confidence Interval, Model 1: socio‐demographic factors (age, sex, education and living situation), Model 2: Model 1 + cognition and dementia diagnosis, Model 3: Model 2 + cultural participation, neighbourhood trust, green/blue spaces and deprivation in the local area (Index of Multiple Deprivation, IMD), Model 4: Model 3 + depressive symptoms and loneliness.

^*^ *p* < 0.05.

^**^ *p* < 0.01.

^***^ *p* ≤ 0.001.

#### 3.2.2 Baseline

At baseline higher education, higher cognitive test scores, higher perceived trust in the local community, greater cultural engagement and perceiving to live near more green and blue spaces were linked with less social isolation (Table 4, models 1–3). Following the addition of loneliness and depression (model 4), higher levels of loneliness (−1.14, 95% CI ‐1.37, −0.90; *p* < 0.001) were associated with greater social isolation, whilst cognitive test scores (0.13; 95% CI 0.04, 0.22; *p* = 0.005), perceived neighbourhood trust (−0.98; 95% CI ‐1.74, −0.22; *p* = 0.012), cultural participation (0.27; 95% CI 0.21, 0.33; *p* < 0.001) and perceiving to live near more green and blue spaces (0.24; 95% 0.12, 0.36; *p* < 0.001) remained predictive of social isolation at T1 (model 4). Depressive symptoms, dementia diagnosis, sex, living situation and level of deprivation in the local area (IMD) were not associated with social isolation at baseline.

## 4 Discussion

This is one of the first comparative studies to examine the prevalence of both loneliness and social isolation in a large cohort of people with dementia. The prevalence of loneliness increased by 4% and isolation by 3% over our 2‐year follow‐up. The loneliness prevalence estimates at T1 (35.4%) and T3 (39.3%) were within the range, but towards the higher end of the spectrum, of those reported by older people in the general population in Europe and the United States of America. [3] Levels of social isolation reported by people with dementia at 28% were comparable to that of community dwelling older adults estimated in recent global meta‐analysis [4] which estimated a prevelance of 29% (95% CI 21–34) worldwide for studies using the Lubben Social Network Scale. Although the levels are higher than in studies of older adults using the same scale in Switzerland (11%), Germany (20%) and 24% (Japan). [29, 38] We observed no longitudinal risk factors for social isolation and only perceived neighbourhood trust was predictive of loneliness across the 24‐month period. At baseline, T1, higher loneliness scores were related to living alone, being under 65 years old, greater depressive symptoms, smaller social networks/greater isolation and lower neighbourhood trust. Lower social isolation was associated with higher cognition scores, greater cultural participation, access to more blue and green spaces, higher neighbourhood trust and less loneliness cross‐sectionally.

The only factor associated with change in loneliness over time was perceived neighbourhood trust. Studies of loneliness consistently identify neighbour/community trust, operationalised in a variety of ways, with lower levels of loneliness although the majority of this is cross‐sectional. [39, 40, 41] However, we found that although the perceived lack of trust group had greater loneliness at baseline this group had a lower increase in loneliness score between T1 and T3 in comparison with the higher perceived neighbourhood trust group. A previous IDEAL study showed that the proportion of people who were likely to trust their neighbours fell slightly over time and overall neighbourhood trust was lower than the population average. [42] For social isolation we did not identify any factors that were predictive in our longitudinal analysis.

Analysis of risk factors associated with loneliness and social isolation at baseline broadly reflected the existing literature. For loneliness living alone, [19] greater depressive symptoms, [24, 43] greater isolation and lower neighbourhood trust [39] were related to increased loneliness at T1. Our finding that people aged under 65 years old were more likely to be lonely compared with their older peers differs from some but not all studies of older adults in the general population. [27, 44] It may correspond to the proposed framework of norm deviations and loneliness (NoDeL) which suggests that people who deviate from social norms, in this instance, average age of dementia onset, may be at a greater risk of loneliness. [45] This could point to support needs for people with younger onset dementia. Higher cognitive test scores, [12, 13] greater cultural participation, [35] access to more blue and green spaces, [46] higher neighbourhood trust [25] and less loneliness were associated with lower social isolation at baseline. In contrast with previous studies [24, 43] living alone was not associated with social isolation for people with dementia. Indeed a longitudinal study found that people with dementia who lived alone had more social contact with people from other households than people with dementia who lived with others and frequency of social contact declined at a slower rate for people living alone. [47] It could be that as cognition declines people become more isolated as they are less able to keep up contacts or get out and engage in activities with others, or may require more support to do so. We found no association between the level of deprivation in the local area measured using the IMD and either loneliness or social isolation. This could indicate that how people with dementia perceive their local area is more relevant to feelings of loneliness or experiences of social isolation than more objective indicators.

Perceived access to blue and green spaces and engagement with cultural activities are important, as they relate to the characteristics of places where people with dementia live and highlight the role of the environment in shaping their experiences of loneliness and social isolation. [48] Prior research indicated that having an engaged and active lifestyle was a key component of living well for people with dementia [49] and that having access to social events and activities was identified as a way of enabling them to live well in the community. [50] This is further highlighted by research showing that higher levels of social and cultural participation amongst people with dementia were associated with greater quality of life and wellbeing in comparison to people with low social and cultural captial. [42] A review and meta‐analysis of technology‐based and social activity interventions highlighted the potential role such interventions might have in reducing loneliness and enhancing social connections and participation in people with dementia. [51].

The lack of similarity of risk factors for loneliness and social isolation reinforces the distinctiveness of these two forms of poor social health. However, the relationship between them is complex as people with dementia who felt lonelier at baseline were more likely to report greater social isolation and vice versa. This raises the possibility of bi‐directional associations suggesting that reducing social isolation may also help address loneliness and vice versa.

It is important to acknowledge the limitations of our approach. As with most longitudinal studies, especially those focussing upon people with dementia, there was notable attrition between timepoints. However, the use of mixed effect models helped to mitigate the impact of this as all participants contributed to intercept estimates and changes in loneliness and social isolation where data was available. Although the IDEAL sample reasonably reflects the UK dementia population attending memory clinic services [52] in Britain we were unable to consider cultural and ethnic differences as the sample was primarily white British. It will be important to consider people with dementia from Black, Asian and minority ethnic groups in future studies of loneliness and social isolation. A recent study showed that people with dementia from ethnic minority groups may experience more loneliness in comparison to white matched peers. [53].

Despite these limitations our study advances understanding of the experience of both loneliness and social isolation for people with dementia. As well as identifying predictors of loneliness and social isolation in this population, we address factors that predict change longitudinally. In the analyses we were able to control for a wider range of explanatory factors than have been explored previously and we included established measures of loneliness and social isolation. Once other factors are considered the mean loneliness and social isolation scores did not increase over our 2‐year follow‐up. Secondly our study highlights that risk factors for these two forms of social ill‐health are distinct both cross‐sectionally and longitudinally. Focussing on longitudinal risk factors neighbourhood trust was predictive of less loneliness. The importance of neighbourhood trust as a factor protective against loneliness is not unique to people with dementia. [41] Consequently, improving neighbourhood trust may address loneliness and isolation for both the general population and people with dementia. This could suggest that if people perceive their local area as more trustworthy then they may feel more able to go out and engage more and maintain their social health. This reminds us that we can improve the well‐being of people with dementia by both specific and generic interventions. It indicates that the characteristics of places where people with dementia live are important contributing factors in their experiences of loneliness and social isolation. [48].

Determining what may increase, or potentially decrease, the risk of loneliness or social isolation in people with dementia may help in the development of screening tools to identify those at risk and inform the design of, and potential targets for, interventions. Our findings extend previous research by suggesting that we may need to differentiate how we address these challenges depending upon whether the focus is on experiencing loneliness or isolation cross‐sectionally or longitudinally. The study also highlights the importance of the local environment and the importance of cultural participation for people with dementia. Initiatives to enhance cultural participation may be beneficial for people with dementia. Enhancing perceptions of support and trust in the local community through initiatives such as dementia friendly communities [1, 50] may also help to reduce experiences of loneliness and social isolation.

## Funding

‘Improving the experience of Dementia and Enhancing Active Life: living well with dementia. The IDEAL study’ was funded jointly by the Economic and Social Research Council (ESRC) and the National Institute for Health and Care Research (NIHR) through grant ES/L001853/2. Investigators: L. Clare, I.R. Jones, C. Victor, J.V. Hindle, R.W. Jones, M. Knapp, M. Kopelman, R. Litherland, A. Martyr, F. Matthews, R.G. Morris, S.M. Nelis, J. Pickett, C. Quinn, J. Rusted, J. Thom. ESRC is part of UK Research and Innovation (UKRI). ‘Improving the experience of Dementia and Enhancing Active Life: a longitudinal perspective on living well with dementia. The IDEAL‐2 study’ was funded by Alzheimer's Society, Grant Nos. 348, AS‐PR2‐16–001. Investigators: L. Clare, I.R. Jones, C. Victor, C. Ballard, A. Hillman, J.V. Hindle, J. Hughes, R.W. Jones, M. Knapp, R. Litherland, A. Martyr, F. Matthews, R.G. Morris, S.M. Nelis, C. Quinn, J. Rusted. This report is independent research supported by the National Institute for Health and Care Research Applied Research Collaboration South‐West Peninsula. The views expressed in this publication are those of the authors and not necessarily those of the ESRC, UKRI, NIHR, the Department of Health and Social Care, the National Health Service, or Alzheimer's Society. The support of ESRC, NIHR and Alzheimer's Society is gratefully acknowledged.

## Ethics Statement

The IDEAL study was approved by the Wales Research Ethics Committee 5 (reference 13/WA/0405) and the Ethics Committee of the School of Psychology, Bangor University (reference 2014–11684). The IDEAL study is registered with UKCRN, registration number 16593.

## Conflicts of Interest

The authors declare no conflicts of interest.

## Data Availability

The data that support the findings of this study are openly available in UK Data Service at http://reshare.ukdataservice.ac.uk/854293/, reference number 10.5255/UKDA‐SN‐854293.
